# Synthesis and Biomedical Applications of Self-healing Hydrogels

**DOI:** 10.3389/fchem.2018.00449

**Published:** 2018-10-02

**Authors:** Yi Liu, Shan-hui Hsu

**Affiliations:** ^1^Institute of Polymer Science and Engineering, National Taiwan University, Taipei, Taiwan; ^2^Institute of Cellular and System Medicine, National Health Research Institutes, Miaoli, Taiwan

**Keywords:** self-healing hydrogel, synthesis mechanism, reversible crosslink, biomedical application, animal model

## Abstract

Hydrogels, which are crosslinked polymer networks with high water contents and rheological solid-like properties, are attractive materials for biomedical applications. Self-healing hydrogels are particularly interesting because of their abilities to repair the structural damages and recover the original functions, similar to the healing of organism tissues. In addition, self-healing hydrogels with shear-thinning properties can be potentially used as the vehicles for drug/cell delivery or the bioinks for 3D printing by reversible sol-gel transitions. Therefore, self-healing hydrogels as biomedical materials have received a rapidly growing attention in recent years. In this paper, synthesis methods and repair mechanisms of self-healing hydrogels are reviewed. The biomedical applications of self-healing hydrogels are also described, with a focus on the potential therapeutic applications verified through *in vivo* experiments. The trends indicate that self-healing hydrogels with automatically reversible crosslinks may be further designed and developed for more advanced biomedical applications in the future.

## Introduction

Hydrogels are constructed by the crosslinked polymer networks as water-swollen gels. Hydrogels have received significant attention as the extracellular matrix mimics for biomedical applications because of their water-retention abilities, appropriate elasticities, and network structures (Wang and Heilshorn, 2015). The self-healing properties, originated from phenomena of wound healing in organisms, are used to describe materials with the ability to restore the morphology and mechanical properties after repeated damages. The microcapsule-laden hydrogels were developed that released healing agents at damage sites (White et al., 2001; Toohey et al., 2007). However, the irreversible healing process and potential interference of fillers limited their applications (Bergman and Wudl, 2008; Syrett et al., 2010). Besides, many dynamic hydrogels typically relied on external stimuli, such as high temperature, low pH, and light, to trigger dynamic crosslinks (Murphy and Wudl, 2010; Harada et al., 2014). The external stimuli would have adverse effects on the cells and living tissues. In this review, self-healing hydrogels are referred that automatically and reversibly repair the damages and recover the functions.

Self-healing hydrogels can be prepared through dynamic covalent bonds and non-covalent interactions. The dynamic equilibrium between dissociation and recombination of various interactions leads the hydrogel to heal damages and reform shapes. Commonly, dynamic covalent bonds exhibit stable and slow dynamic equilibriums, while non-covalent interactions show fragile and rapid dynamic equilibriums (Zou et al., 2017). With versatile mechanical properties, self-healing hydrogels can be manufactured with robust, shear-thinning, or cell-adaptable properties for a broad range of applications, such as soft robots, 3D printing, and drug/cell delivery. In this review paper, we will take a detailed look at the current synthesis and biomedical applications of self-healing hydrogels. Firstly, various advanced strategies are introduced about the preparations and the mechanisms of self-healing hydrogels. Subsequently, biomedical applications of the self-healing hydrogels are described, especially, the ones that have been evaluated by animal models.

## Self-healing mechanisms

**Figure 1:**
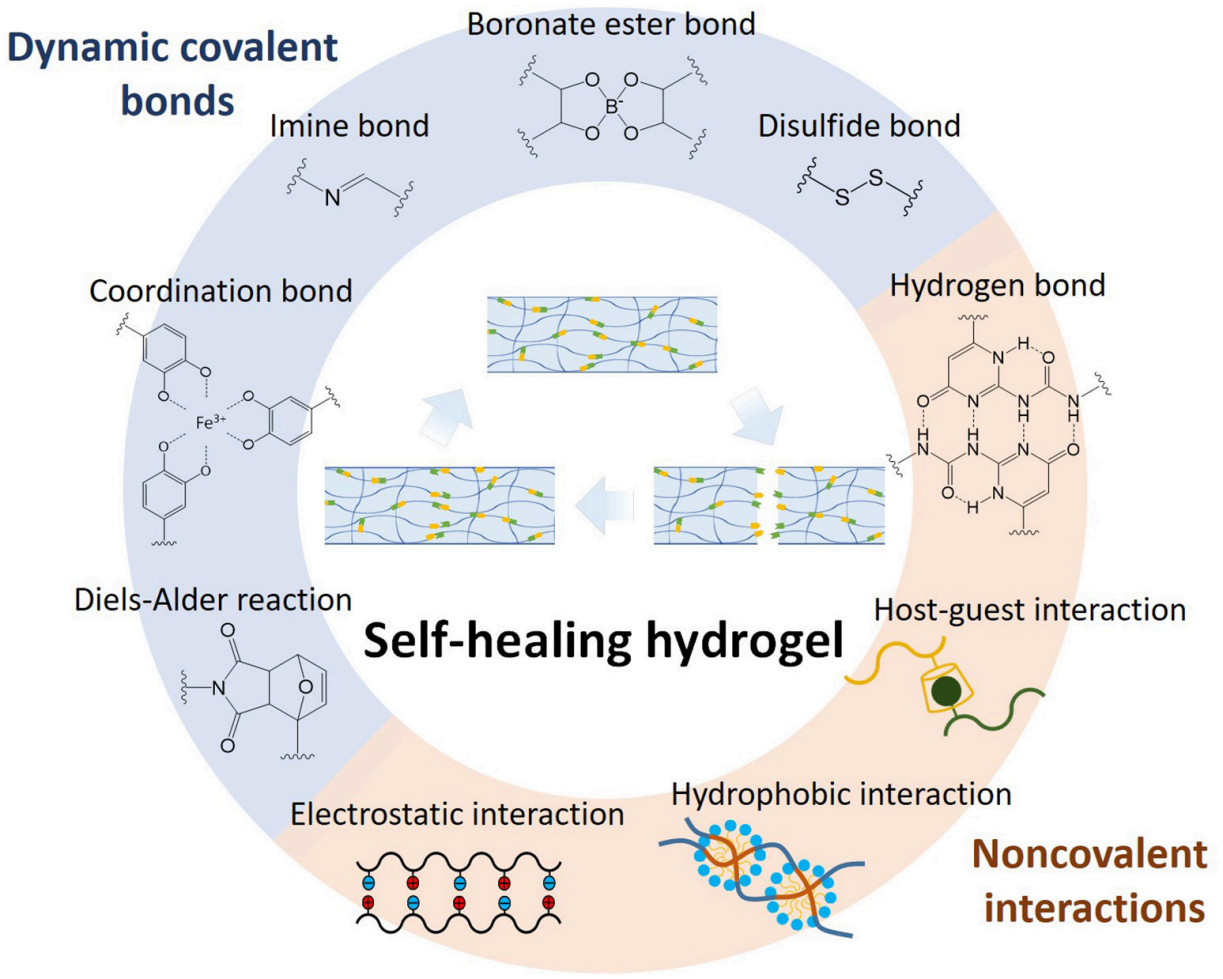
Self-healing chemistries and mechanisms for various self-healing hydrogels, including dynamic covalent bonds, non-covalent interactions, and multi-mechanism interactions.

Self-healing hydrogels have been synthesized based on different chemistries and mechanisms as shown in Figure 1, including dynamic covalent bonds, non-covalent interactions, and multi-mechanism interactions. Each will be elaborated below.

### Dynamic covalent bonding

Dynamic covalent chemistry, including imine formation, boronate ester complexation, catechol-iron coordination, Diels-Alder reaction, and disulfide exchange, is widely applied in the formation of self-healing hydrogel. Dynamic covalent bonds exhibit the stronger but slower dynamic equilibrium compared to non-covalent interactions.

The imine (or referred as Schiff base) is a compound with a carbon-nitrogen double bond formed by nucleophilic attack of amine to aldehyde or ketone. A number of self-healing hydrogels have been developed by aliphatic Schiff bases (Lü et al., 2015; Zhu, D. et al., 2017; Huang et al., 2018) or aromatic Schiff bases (Karimi and Khodadadi, 2016; Qu et al., 2017), in which aromatic Schiff bases show higher stability to maintain the mechanical properties compared to aliphatic Schiff bases (Zhang et al., 2011). Zhang et al. synthesized a dibenzaldehyde-terminated telechelic poly(ethylene glycol), namely difunctionalized PEG (DF-PEG), to form self-healing hydrogel through aromatic Schiff bases between benzaldehyde groups of DF-PEG and amino groups of chitosan (Zhang et al., 2011). The hydrogels were prepared rapidly under mild conditions at 20°C within 60 s, and they could be degraded by acidic pH, amino acids, vitamin B6 derivatives, and enzymes. The hydrogels were developed for 3D cell culture and cell delivery due to their cytocompatibility and injectability (Yang et al., 2012; Li et al., 2017; Zhang, Y. L. et al., 2017). Acylhydrazone and oxime are derivatives of imine with great stability, which have also been developed to synthesize the self-healing hydrogels (Deng et al., 2010; Grover et al., 2012; Lin et al., 2013; Mukherjee et al., 2015). For example, the self-healing hydrogel was prepared by adding oxidized sodium alginate into the mixture of N-carboxyethyl chitosan and adipic acid dihydrazide via dynamic imine and acylhydrazone bonds (Wei et al., 2015).

The reversible boronate ester bond is formed by complexation of boronic acid and diol, and its stability is dependent on pH-value and glucose concentration. Boronic acid and its derivatives, such as phenylboronic acid or phenylboronic acid-incorporated polymers, have been widely developed to prepare self-healing hydrogels. Yesilyurt et al. mixed phenylboronic acid and diol-modified poly(ethylene glycol) to form self-healing hydrogel that exhibited pH-responsive tunable mechanical properties, and glucose-responsive size-dependent release of proteins (Yesilyurt et al., 2016). The hydrogel was cytocompatible *in vitro*, and it showed a typical foreign body reaction *in vivo* without chronic inflammation. He et al. prepared self-healing hydrogel via complexation of a catechol-modified polymer and 1,3-benzenediboronic acid, which demonstrated high stability under alkaline conditions and low stability under acidic conditions (He et al., 2011). Another self-healing hydrogel was fabricated using the mixture of poly(ethylene glycol) diacrylate, dithiothreitol, and borax via permanent thiol-ene Michael addition and dynamic borax diol complexation in one-pot approach (He et al., 2015). The hydrogel can be injected with cells to form branched tubular channels for vascularization *in vitro* and easily removed by immersion in cell culture medium (Tseng et al., 2017).

The reversible coordinate bond between catechol and iron has been developed to prepare self-healing hydrogels. The reversibility of catechol-iron coordination bond can be controlled by adjusting pH conditions (Krogsgaard et al., 2013). When the environmental pH is raised from acidic to basic values, a rapidly self-healing hydrogel with high strength may form. Li et al. incorporated iron oxide (Fe_3_O_4_) nanoparticles with the catechol-modified polymers to form a self-healing hydrogel via reversible coordination bonds at the nanoparticle surface (Li et al., 2016). Self-healing hydrogel based on catechol-Fe_3_O_4_ nanoparticles structures exhibited magnetic properties and solid-like mechanics, in comparison with the fluid-like hydrogel by catechol-Fe(III) crosslinking.

The disulfide exchange provides dynamic covalent bonds to form self-healing hydrogels, which are sensitive to pH or redox potential (Wei, Z. et al., 2014). Recently, 1,2-dithiolane-functionalized polymers were synthesized to form self-healing hydrogels with rapid sol-gel transition via the disulfide exchange between the 1,2-dithiolane and dithiols (Barcan et al., 2015; Yu et al., 2017; Zhang and Waymouth, 2017). The disulfide exchange of 1,2-dithiolane can reform under neutral or weakly alkaline conditions, which can be further controlled by temperature.

Another important dynamic covalent chemistry in self-healing hydrogels is the thermally reversible Diels-Alder reaction (Liu and Chuo, 2013; Zhao et al., 2016; Shao et al., 2017). However, the biomedical applications of Diels-Alder reaction are limited because Diels-Alder bonds need a high temperature and a long duration to cleave and reform for self-healing properties. In recent reports, Diels-Alder chemistry was developed to form self-healing hydrogels combining with other reversible interactions, such as electrostatic interaction (Banerjee and Singha, 2017; Ghanian et al., 2018), coordination bond (Li et al., 2018a), imine bond (Li et al., 2018b), and acylhydrazone bond (Yu et al., 2015).

### Non-covalent interactions

Self-healing hydrogels can be produced through non-covalent interactions, such as hydrogen bond, electrostatic interaction, and hydrophobic interaction. The non-covalent interactions are less stable and more sensitive to environmental conditions (such as pH and temperature) compared to covalent interactions. However, robust self-healing hydrogels can still form based on non-covalent interactions via special manufacturing procedures or nano- and micro-structures.

The hydrogen bonding is an attractive interaction between the hydrogen atoms and electronegative atoms, in which the hydrogen atom is bound to a high electronegative atom, such as nitrogen, oxygen, and fluorine. The polyvinyl alcohol-based self-healing hydrogels were developed using the freezing/thawing method via hydrogen bonding (Zhang et al., 2012; Zhang, Z. et al., 2017). Moreover, hydrogen bonding-based self-healing hydrogels were frequently reported with incorporations of various chemical moieties, such as 2-ureido-4-pyrimidone (UPy) moieties (Cui and del Campo, 2012; Dankers et al., 2012; Cui et al., 2013; Bastings et al., 2014; Chirila et al., 2014; Hou et al., 2015; Zhang et al., 2016), nucleobase moieties (Ye et al., 2017), deferoxamine moieties (Xu et al., 2017), and gallol moieties (Shin and Lee, 2017). In a recent work, the cytosine- and guanosine-modified hyaluronic acid (HA) formed self-healing hydrogel by Watson-Crick base pairing between the nucleobases through hydrogen bonding (Ye et al., 2017). The hydrogel exhibited pH-stimulated sol-gel transition where the hydrogel exhibited gel state in pH 6–8 and sol state in pH < 6 or > 8. In another example, Shin and Lee synthesized gallol-conjugated HA and added a gallol-rich crosslinker (i.e., oligo-epigallocatechin gallate) to form a shear-thinning and self-healing hydrogel based on extensive hydrogen bonds of gallol-gallol and gallol-HA (Shin and Lee, 2017). The hydrogel was resistant to enzymatic degradation by protein (i.e., hyaluronidase) immobilization through non-covalent interactions between gallols and proteins.

Hydrophobic interactions occur as a consequence of aggregative hydrophobes in aqueous media. In many cases of self-healing hydrogels based on hydrophobic interactions, the surfactant micelles (Gulyuz and Okay, 2015; Liu, Y. et al., 2018) or liposomes (Rao et al., 2011; Hao et al., 2013) are employed as crosslinking points to construct the polymer chains comprising both hydrophilic and hydrophobic monomers. For example, the self-healing hydrogel could form via micellar copolymerization of hydrophobic monomer stearyl methacrylate and hydrophilic monomer acrylamide in the aqueous solution of sodium dodecyl sulfate (SDS) micelles (Tuncaboylu et al., 2011, 2012a,b). In these cases, the addition of salt into aqueous SDS solutions leads to micellar growth and solubilization of hydrophobes within SDS micelles. The hydrogel containing SDS micelles with the time-dependent dynamic moduli exhibited high elongation ratio and good self-healing ability, while after extraction of SDS, the hydrogel with time-independent dynamic moduli showed high mechanical strength and no self-healing ability. Self-healing hydrogel can also be prepared based on surfactant-free hydrophobic associations via solvent evaporation of an aqueous polymer solution above a critical polymer concentration (Owusu-Nkwantabisah et al., 2017).

Self-healing hydrogels can form through reversible electrostatic interactions occurring in charged polymers and ions (Wei et al., 2013; Wei, H. et al., 2014), polyelectrolytes (Huang et al., 2014; Luo et al., 2015; Ren et al., 2016; Li, J. et al., 2017), polyampholytes (Ihsan et al., 2013; Sun et al., 2013), and zwitterionic fusions (Bai et al., 2014). For example, self-healing hydrogel was synthesized through reversible polyelectrolyte complexes of alginate and 2-hydroxypropyltrimethyl ammonium chloride chitosan (Ren et al., 2016). The two polymers were mixed to form self-healing hydrogel at charge neutrality followed by precipitation for 12 h. In addition to self-healing ability, the hydrogel exhibited shear-thinning property, high adhesive behavior, and cytocompatibility. Meanwhile, the polyampholytes can form self-healing hydrogels with tunable mechanical properties via electrostatic interactions between randomly dispersed cationic and anionic repeating groups in polymers (Sun et al., 2013). In analogy to double-network hydrogels, the tough polyampholyte hydrogels contained ionic strong bonds and weak bonds to maintain the shapes and enhance the shock absorbance and self-healing abilities, respectively. Besides, the more hydrophobic polyampholyte hydrogels exhibited the robust and poor self-healing properties, whereas the less hydrophobic polyampholyte hydrogels exhibited the soft and good self-healing properties (Sun et al., 2013).

### Multi-mechanism interactions

Supramolecular chemistry is widely applied to prepare self-healing hydrogels through various non-covalent interactions, such as host–guest interaction and protein–ligand recognition. In addition, hybrids of non-covalent interactions and/or permanent/dynamic covalent bonds were developed to prepare self-healing hydrogels for rapid recovery, long-term stability, high mechanical property, and/or multi-responsive behavior.

Host–guest interactions occur when two or more chemical species assemble via non-covalent interactions, such as van der Waals force, hydrogen bond, electrostatic interaction, and hydrophobic interaction. In host–guest chemistry, the macrocyclic host moiety is inserted inside the guest moiety to form a unique structure of the inclusion complexation. Host–guest interactions are used popularly to prepare the self-healing hydrogels, and many such hydrogels rely on external stimuli, such as temperature (Zheng et al., 2012), light (Yamaguchi et al., 2012), pH (Zheng et al., 2013), and redox potentials (Nakahata et al., 2011; Miyamae et al., 2015), to trigger the healing process. Meanwhile, host–guest hydrogels have also been developed to recover themselves without the need of external stimuli (Appel et al., 2012; Kakuta et al., 2013; Rodell et al., 2013; McKee et al., 2014). For example, the self-healing HA hydrogel was prepared based on the host–guest interactions of β-cyclodextrin-modified HA (host macromer) and adamantane-modified HA (guest macromer) (Rodell et al., 2013). The hydrogels exhibited shear-thinning property and rapid recovery at 25°C.

Catechol and gallol are polyphenolic moieties commonly distributed in organisms as important functional groups, which can form various covalent and non-covalent bonds, such as Michael addition or Schiff base reaction with thiol and amine, coordination bonds with metals, hydrogen bonds, and aromatic interactions (Lee et al., 2007; Sileika et al., 2013). Li et al. developed a novel self-healing hydrogel by self-assembly of an ABA tri-block copolymer through the catechol-mediated hydrogen bonding and aromatic interaction, where the catechol-functionalized poly(N-isopropylacrylamide) (PNIPAM) and poly(ethylene oxide) (PEO) were each selected as A and B blocks for synthesis (Li et al., 2015). The hydrogel exhibited a thermo-responsive sol-gel transition and recovered its mechanical properties after repeated damages owing to PNIPAM moiety and catechol-mediated interaction, respectively. Moreover, Birkedal and coworkers prepared a self-healing and pH-responsive hydrogel using tannic acid (TA), metal ions, and polyallylamine (PAA) in one step (Krogsgaard et al., 2014a). Below pH 8, the hydrogel was crosslinked mostly by reversible hydrogen bonds, covalent crosslinks between TA and PAA, and coordination bonds between TA and iron ion; while above pH 8, irreversible bonds predominantly enhanced the gel modulus and hindered self-healing. Likewise, self-healing hydrogels were developed based on interactions between 3,4-dihydroxyphenylalanine-modified PAA (DOPA-PAA) and metal ions [such as Al(III), Ga(III), In(III), and Fe(III) ions; (Krogsgaard et al., 2014b)].

Dupin and coworkers reported for the first time the formation of self-healing hydrogels using gold(I) ions-crosslinked thiol-terminated PEG via metallophilic attractive forces (Casuso et al., 2014). The hydrogel exhibited cytocompatibility and mimicked the synovial fluid of the human joint in rheological properties under physiological conditions. Afterward, a series of self-healing hydrogels with tunable mechanical properties were prepared using HAuCl_4_ (or AgNO_3_) and 4-arm thiol-terminated polyethylene glycol [(PEGSH)_4_] in different ratios based on metal(I)-thiolate/disulfide exchange (Casuso et al., 2015). These hydrogels showed reversible mechanical properties and frequency-dependent stiffness/shock-absorbing properties at the physiological pH due to the metal(I)-thiolate/disulfide exchange. The potential of the hydrogel as an artificial nucleus pulposus for the intervertebral discs was demonstrated via a bovine *ex vivo* model using axial compression-tension cycles at different frequencies followed by creep experiments and μCT analysis (Pérez-San Vicente et al., 2017). Moreover, the hydrogels incorporating bioactive glass nanoparticles led to the stiffer properties for bone regeneration (Gantar et al., 2016). Meanwhile hydroxyapatite was formed after degradation of the nanoparticles.

On the basis of dynamic acylhydrazone and disulfide bonds, self-healing hydrogels with pH/redox dual responsive transitions have been developed (Deng et al., 2012). The hydrogel displayed self-healing properties in acidic and basic conditions based on the acylhydrazone and disulfide bonds, respectively. Additionally, acylhydrazone bonds were activated by the catalytic aniline in neutral conditions, and disulfide bonds were responsive to the redox conditions. Recently, the self-healing hydrogel was prepared from the mixture carboxyethyl cellulose-graft-dithiodipropionate dihydrazide and DF-PEG under 4-amino-DL-phenylalanine (4a-Phe) catalysis (Yang et al., 2017). The gelation time of the hydrogel could be controlled by varying the total polymer content or the 4a-Phe concentration. The hydrogel was applied for controlled release of doxorubicin and 3D culture of L929 cells because of pH/redox responsiveness and cytocompatibility.

## Biomedical applications of self-healing hydrogels

Self-healing hydrogels have received increasing attentions in biomedical applications, such as wound healing (Gaharwar et al., 2014; Han et al., 2016; Zhao et al., 2017; Zhu, S. K. et al., 2017; Li et al., 2018; Liu, B. et al., 2018), drug delivery (Huebsch et al., 2014; Liu et al., 2016; Wang et al., 2016; Xing et al., 2016; Wang J. Y. et al., 2017; Xia et al., 2017; Yavvari et al., 2017; Zhu, C. et al., 2017; Hong et al., 2018), tissue engineering (Dankers et al., 2012; Bastings et al., 2014; Gaffey et al., 2015; Rodell et al., 2015a,b; Loebel et al., 2017), surface coating (Canadell et al., 2011; Yoon et al., 2011; Yang et al., 2015), 3D printing (Highley et al., 2015; Darabi et al., 2017; Loebel et al., 2017; Wang et al., 2018), and soft robot (Shi et al., 2015; Darabi et al., 2017; Han et al., 2017; Liu, B. et al., 2018; Liu et al., 2018). In these cases, dibenzaldehyde-based, UPy-based, catechol-based, and host–guest-based self-healing hydrogels are highlighted due to many evaluations of *in vivo* experiments. As summarized in Table 1, some animal models have been used to verify the biocompatibility and efficacy of self-healing hydrogels. Besides the biocompatibility, self-healing hydrogels require injectability and long-term stability for drug delivery, tissue engineering, and 3D printing; and toughness and conductivity for soft robot.

**Table 1:** Examples of self-healing hydrogels evaluated by animal models.

**Self-healing mechanisms**	**Materials**	**Animal model evaluation**	**References**
Boronate ester bonds	Alginate-boronic acid	Oral administration for drug retention	Hong et al., 2018
Coordination bonds	Dexamethasone phosphate and Ca(II)	Subcutaneous injection for drug delivery	Liu et al., 2016
Coordination bonds	Chitosan-catechol and Fe(III)	Cancer model for drug delivery	Yavvari et al., 2017
Coordination bonds and electrostatic interactions	Collagen and gold	Cancer model for drug delivery	Xing et al., 2016
Electrostatic interactions	Silicate nanoplatelets and gelatin	Liver bleeding model for hemostasis	Gaharwar et al., 2014
Hydrogen bonds	Polyglutamic acid and lysine	Skin defect model for wound healing	Zhu, S. K. et al., 2017
Hydrogen bonds	Ureidopyrimidinone-PEG	Kidney implantation for tissue repair	Dankers et al., 2012
Hydrogen bonds	Ureidopyrimidinone-PEG	Myocardial infarction model for tissue repair	Bastings et al., 2014
Hydrogen bonds	Gelatin methacrylate and tannic acid	Gastric incision model for wound closure	Liu, B. et al., 2018
Hydrogen bonds and aromatic interactions	Polydopamine nanoparticles and poly(N-isopropylacrylamide)	Skin defect model for wound healing	Han et al., 2016
Hydrogen bonds and aromatic interactions	Polydopamine, graphene oxide, and polyacrylamide	Osteochondral defect model for tissue repair	Han et al., 2017
Host-guest interactions	β-Cyclodextrin-PEI and adamantane-PEG	Myocardium injection for drug delivery	Wang L. L. et al., 2017
Host-guest interactions	Adamantane/thiol-HA and cyclodextrin/methacrylate-HA	Myocardial infarction model for tissue repair	Rodell et al., 2015a
Host-guest interactions	Adamantane-HA and β-cyclodextrin-HA	Myocardial infarction model for tissue repair	Gaffey et al., 2015
Host-guest interactions	Adamantane-HA and β-cyclodextrin-HA	Chronic kidney disease model for drug delivery	Rodell et al., 2015b
Imine bond	DF-PEG and chitosan-aniline tetramer	Subcutaneous injection for cell retention	Dong et al., 2016
Imine bond	Chondroitin sulfate-aldehyde and N-succinyl-chitosan	Subcutaneous injection for material degradation	Lü et al., 2015
Imine bond	DF-PEG-co-poly(glycerol sebacate) and chitosan-polyaniline	Skin defect model for wound healing	Zhao et al., 2017
Imine bond	Aldehyde-xanthan and carboxymethyl-chitosan	Abdominal wall defect model for tissue repair	Huang et al., 2018
Imine bond	DF-PEG and glycol chitosan	Cancer model for drug delivery	Xia et al., 2017
Imine bond	DF-PEG-co-poly(glycerol sebacate) and Chitosan-polyaniline	Liver bleeding model for hemostasis	Zhao et al., 2017
Imine bond	DF-PEG, glycol chitosan, fibrinogen, and thrombin	Hindlimb ischemia model for tissue repair	Hsieh et al., 2017
Imine bond	DF-PEG and glycol chitosan	Zebrafish neural injury model for tissue repair	Tseng et al., 2015
Imine bond	DF-PEG and glycol chitosan	Zebrafish neural injury model for drug delivery	Hsieh et al., 2018
Imine bond	DF-PEG, glycol chitosan, fibrinogen, and thrombin	Zebrafish embryos injection for angiogenesis	Hsieh et al., 2017

### Drug delivery

Self-healing hydrogel based on host–guest interactions between β-cyclodextrin-modified PEI and adamantane-modified PEG was developed for local siRNA release (Wang et al., 2018). The modified polymers assembled with siRNA to form polyplexes, which could improve the transfection efficiency and the viability of cells. When injected into the myocardium, the hydrogel with siRNA encapsulation enhanced the uptake of Cy5.5-siRNA and maintained the silencing of GFP for 1 week in a GFP-expressing rat.

Xing et al. reported an injectable and self-healing collagen-gold hybrid hydrogel with adjustable mechanical properties (Xing et al., 2016). This hydrogel was prepared through electrostatic interaction between positively charged collagen chains and negatively charged tetrachloroaurate ([AuCl_4_]^−^) ions, and further non-covalent interactions between subsequent biomineralized gold nanoparticles and collagen. The hydrogel was developed for localized delivery and sustained release of the photosensitive drug. By combinatorial photothermal and photodynamic therapies, the significantly enhanced antitumor efficacy was demonstrated through an *in vivo* antitumor test using the subcutaneous mouse model.

Self-healing hydrogels based on glycol chitosan and DF-PEG (GC-DP) have been developed for intratumor therapy *in vivo*. GC-DP hydrogel containing antitumor drug was injected into the disease position with a steady release *in situ* (Yang et al., 2017). Moreover, the ionic GC-DP hydrogel exhibited microwave susceptibility to produce high-temperature hyperthermia for tumor ablation (Wang J. Y. et al., 2017). A multi-antitumor system was developed based on GC-DP hydrogel containing doxorubicin/docetaxel-loaded poly(lactic-co-glycolic acid) (PLGA) nanoparticles and iron oxide for chemotherapy and magnetic hyperthermia (Xie et al., 2017). The system showed the greater *in vivo* antitumor efficacy under the alternative magnetic field compared to the hydrogel containing doxorubicin/docetaxel-loaded PLGA nanoparticles.

### Tissue engineering

Self-healing host–guest hydrogels have been developed to treat the myocardial infarction. The self-healing hydrogel, formed through host–guest interactions of adamantine- and β-cyclodextrin-modified HA, was injected into the ischemic myocardium encapsulating endothelial progenitor cells (EPCs) (Gaffey et al., 2015). A rodent model of acute myocardial infarction was employed to confirm that a significant increase in vasculogenesis was noted with the hydrogel encapsulating EPCs, compared to the treatment of EPCs alone or hydrogel alone. Moreover, the hydrogel was designed using adamantane/thiol-modified HA and cyclodextrin/methacrylate-modified HA through host–guest interaction and Michael addition (Rodell et al., 2015a). The reversible host–guest interaction and permanent Michael addition provided shear-thinning injection and high retention, respectively. Epicardial injection of the hydrogel in a rat myocardial infarction model showed significant improvement of the outcome compared to the untreated group and the hydrogel without Michael addition.

Self-healing hydrogels were designed as injectable carriers for growth factors using PEG end-functionalized with four-fold hydrogen-bonding ureidopyrimidinone (UPy) moieties. UPy-modified PEG hydrogel incorporated with antifibrotic growth factor was delivered in a pocket introduced under the kidney capsule of rats (Dankers et al., 2012). The kidney capsule was loosened from the kidney to create a small pocket. After injection of growth factor-containing hydrogels, the number of myofibroblasts stayed the same to the contralateral (healthy) kidney, while significantly increased with the injection of saline or hydrogel alone. In another example, growth factors were delivered by UPy-modified PEG hydrogel to repair the infarcted myocardium (Bastings et al., 2014). This pH-switchable hydrogel could be injected through the long and narrow lumen of the catheter mapping system, and rapidly formed a hydrogel in contact with tissue. The growth factor-containing hydrogel reduced scar collagen in a chronic myocardial infarction pig model.

Self-healing hydrogels based on GC-DP was prepared for tissue repairs. In the application of central nervous system (CNS) repair, neurosphere-like progenitors showed better proliferation and differentiation in GC-DP hydrogel, and injection of GC-DP hydrogel combining neurospheres promoted functional recovery in a zebrafish CNS impaired model (Tseng et al., 2015). Moreover, the GC-DP hydrogel combining the optogenetic method was developed as a temporal-spatial approach to treat neurodegenerative diseases (Hsieh et al., 2018). The hydrogel containing bacteriorhodopsin plasmid and neural stem cells was injected into CNS impaired zebrafish where the neural repair was observed, particularly under green light exposure. Besides, GC-DP hydrogel was also used to induce blood capillary formation. With the incorporation of fibrin gel, a composite hydrogel could form with an interpenetrating polymer network (i.e., double network) of GC-DP and fibrin (Hsieh et al., 2017). The hydrogel induced vascular endothelial cells to form capillary-like structures, and injection of the hydrogel alone promoted angiogenesis in zebrafish and rescued the blood circulation in ischemic hindlimbs of mice.

### Other applications

Self-healing hydrogels, based on the host–guest interaction of β-cyclodextrin- and adamantine-modified HA, were used in the 3D printing of high-resolution structures through printing of shearing-thinning ink hydrogel into self-healing support hydrogel (Highley et al., 2015). The multicellular structures could be expediently patterned, such as printing of mesenchymal stem cells within an ink hydrogel into a support hydrogel containing 3T3 fibroblasts. The channel-like structure was achieved by writing the ink hydrogel into the methacrylate-modified support hydrogel, followed by UV irradiation for secondary covalent crosslinks of support hydrogel, followed by removal of the physical (i.e., host–guest) ink hydrogel. Meanwhile, the self-supporting structure was obtained by covalently crosslinking the ink hydrogel and removing the non-covalent support hydrogel. This system supported the patterning of multiple inks, cells, and channels in 3D space.

A tough self-healing hydrogel was synthesized as cell stimulators and implantable bioelectronics (Han et al., 2017). In the study, graphene oxide was partially converted to conductive graphene through polydopamine reduction, and acrylamide monomers were polymerized *in situ* to form the hydrogel by interactions between graphene oxide, polydopamine, and polyacrylamide. Meanwhile, the free catechol groups on polydopamine imparted self-healing property and tissue adhesion to the hydrogel via various non-covalent interactions. The hydrogel could be used not only as an adhesive electrode or motion sensor but also as an *in vitro* cell stimulator and *in vivo* implantable intramuscular electrode. For example, the hydrogel electrodes were implanted into the rabbit dorsal muscle and connected to a signal detector using the transcutaneous wires. The electrodes could record the electromyographic signal when the rabbit was interfered with external stimulation.

## Conclusions

Self-healing hydrogels can be classified as robust and soft hydrogels according to mechanical properties in biomedical applications. Robust self-healing hydrogels are used as soft robots (such as implantable or wearable biosensors) with extended lifetime and mechanical performance due to repairing of the damages or fatigues. Soft self-healing hydrogels with shear-thinning properties are used in cell/drug delivery and 3D printing due to injection through narrow needles and retention at target sites. To facilitate biomedical applications in the future, self-healing hydrogels need to address several major concerns including (1) designing self-healing hydrogels with good biocompatibility and appropriate mechanical properties; (2) better characterizing the self-healing properties with various assessment tools (such as rheological measurement, mechanical analysis, or other novel tools); (3) developing theories on self-healing mechanisms and properties (such as chemistry, kinetics, and thermodynamics); and (4) translation by animal experiments and clinical trials. Moreover, because the self-healing properties of hydrogels are mostly determined in non-physiological environments, it would be challenging to verify that the known self-healing properties are well-maintained in physiological conditions such as with electrolytes, under mechanical stress, and in the presence of material–cell interaction. In addition, controllable biodegradability is important in self-healing hydrogels for tissue engineering and drug delivery. In comparison with permanent crosslinks, reversible crosslinks are broken easily to facilitate biodegradation, while reversible crosslinks recover the macro- and micro-scaled damages to restrain biodegradation. Reversible equilibriums of self-healing hydrogels should be controlled according to the various applications, such as long-term drug release and cell-adaptable materials.

## Author contributions

All authors listed have made a substantial, direct and intellectual contribution to the work, and approved it for publication.

### Conflict of interest statement

The authors declare that the research was conducted in the absence of any commercial or financial relationships that could be construed as a potential conflict of interest.
